# Benefits of Paediatric to Adult Transition Programme in Inflammatory Bowel Disease: The BUTTERFLY Study of GETECCU and SEGHNP

**DOI:** 10.3390/jcm12144813

**Published:** 2023-07-21

**Authors:** Cristina Rubín de Célix, Javier Martín-de-Carpi, Gemma Pujol-Muncunill, Laura María Palomino, Marta Velasco Rodríguez-Belvís, Rafael Martín-Masot, Víctor Manuel Navas-López, Elena Ricart, María José Casanova, Alejandro Rodríguez-Martínez, Eduardo Leo-Carnerero, Alba Alcaraz, Miriam Mañosa, Vicent Hernández, María Consuelo Cobelas Cobelas, César Sánchez, Luis Menchén, Francisco Mesonero, Manuel Barreiro-De Acosta, Nazareth Martinón-Torres, Coral Tejido Sandoval, Alicia Rendo Vázquez, Pilar Corsino, Raquel Vicente, Alejandro Hernández-Camba, José Ramón Alberto Alonso, I. Alonso-Abreu, Ana María Castro Millán, Laia Peries Reverter, Beatriz Castro, Estela Fernández-Salgado, M. Mercedes Busto Cuiñas, José Manuel Benítez, Lucía Madero, Fernando Clemente, Sabino Riestra, Santiago Jiménez-Treviño, Maia Boscá-Watts, Elena Crehuá-Gaudiza, Marta Calvo Moya, José María Huguet, Ester-María Largo-Blanco, Leticia González Vives, Rocío Plaza, Iván Guerra, Josefa Barrio, Laura Escartín, Erika Alfambra, Noelia Cruz, M. Carmen Muñoz, María Guadalupe Muñoz Pino, Manuel Van Domselaar, Belén Botella, David Monfort Miquel, M. Carmen Rodríguez Grau, Agustín De La Mano, Yolanda Ber, María Calvo Iñiguez, Teresa de Jesús Martínez-Pérez, María Chaparro, Javier P. Gisbert

**Affiliations:** 1 Gastroenterology Department, Hospital Universitario de La Princesa, Instituto de Investigación Sanitaria Princesa (IIS-Princesa), Universidad Autónoma de Madrid (UAM), Centro de Investigación Biomédica en Red de Enfermedades Hepáticas y Digestivas (CIBEREHD), 28006 Madrid, Spain; 2 Department of Paediatric Gastroenterology, Hepatology and Nutrition, Hospital Sant Joan de Déu, 08950 Barcelona, Spain; 3 Paediatric Gastroenterology and Nutrition Department, Hospital Infantil Universitario Niño Jesús, 28009 Madrid, Spain; 4 Paediatric Gastroenterology and Nutrition Unit, Hospital Regional Universitario de Málaga, Biomedical Re-search Institute of Málaga (IBIMA), 29010 Málaga, Spain; 5 Gastroenterology Department, Hospital Clínic, Centro de Investigación Biomédica en Red de Enfermedades Hepáticas y Digestivas (CIBEREHD), Institut d’Investigacions Biomèdiques Agustí Pi i Sunyer (IDIBAPS), 08036 Barcelona, Spain; 6 Paediatric Gastroenterology, Hepatology and Nutrition, UGC de Pediatría, Hospital Universitario Virgen del Rocío, 41013 Sevilla, Spain; 7 Gastroenterology Department, Hospital Virgen del Rocío, 41013 Sevilla, Spain; 8 Department of Paediatric Gastroenterology, Nutrition and Hepatology, University Hospital Germans Trias I Pujol, 08916 Badalona, Spain; 9 Gastroenterology Department, Hospital Universitari Germans Trias I Pujol, Centro de Investigación Biomédica en Red de Enfermedades Hepáticas y Digestivas (CIBEREHD), 08916 Badalona, Spain; 10 Department of Gastroenterology, Xerencia Xestion Integrada de Vigo, SERGAS, Research Group in Digestive Diseases, Galicia Sur Health Research Institute (IIS Galicia Sur), SERGAS-UVIGO, 36312 Vigo, Spain; 11 Department of Paediatrics, Xerencia Xestion Integrada de Vigo, SERGAS, 36312 Vigo, Spain; 12 Paediatric Gastroenterology, Hepatology and Nutrition Department, Hospital General Universitario Gregorio Marañón, 28018 Madrid, Spain; 13 Gastroenterology Department–CEIMI, Hospital General Universitario Gregorio Marañón, Departamento de Medicina, Universidad Complutense de Madrid, 28018 Madrid, Spain; 14 Gastroenterology Department, Hospital Universitario Ramón y Cajal, 28034 Madrid, Spain; 15 Gastroenterology Department, Hospital Universitario Clínico de Santiago, 15706 Santiago de Compostela, Spain; 16 Paediatric Gastroenterology, Hepatology and Nutrition Unit, Hospital Universitario Clínico de Santiago, 15706 Santiago de Compostela, Spain; 17 Gastroenterology Department, Complejo Hospitalario Universitario de Ourense, 32005 Orense, Spain; 18 Paediatric Gastroenterology, Hepatology and Nutrition Unit, Complejo Hospitalario Universitario de Ourense, 32005 Orense, Spain; 19 Inflammatory Bowel Disease Unit, Gastroenterology Department, Hospital Universitario Miguel Servet, Health Research Institute of Aragón, 50009 Zaragoza, Spain; 20 Gastroenterology Department, Hospital Universitario Nuestra Señora de Candelaria, 38010 Santa Cruz de Tenerife, Spain; 21 Paediatric Gastroenterology, Hepatology and Nutrition Unit, Hospital Universitario Nuestra Señora de Candelaria, 38010 Santa Cruz de Tenerife, Spain; 22 Gastroenterology Department, Hospital Universitario de Canarias, 38320 Santa Cruz de Tenerife, Spain; 23 Paediatric Gastroenterology, Hepatology and Nutrition Unit, Hospital Universitario de Canarias, 38320 Santa Cruz de Tenerife, Spain; 24 Gastroenterology Department, Hospital Universitari de Girona Doctor Josep Trueta, 17007 Girona, Spain; 25 Gastroenterology Department, Hospital Universitario Marqués de Valdecilla, 39008 Santander, Spain; 26 Gastroenterology Department, Complejo Hospitalario Universitario de Pontevedra, 36071 Pontevedra, Spain; 27 Paediatric Gastroenterology, Hepatology and Nutrition Unit, Complejo Hospitalario Universitario de Pontevedra, 36071 Pontevedra, Spain; 28 Gastroenterology Department, Hospital Universitario Reina Sofía, IMIBIC, 14004 Córdoba, Spain; 29 Gastroenterology Department, Hospital Universitario de Alicante, Instituto de Investigación Sanitaria y Biomedica (ISABIAL), 03010 Alicante, Spain; 30 Paediatric Gastroenterology, Hepatology and Nutrition Unit, Hospital Universitario de Alicante, 03010 Alicante, Spain; 31 Gastroenterology Department, Hospital Universitario Central de Asturias, Instituto de Investigación Sanitaria del Principado de Asturias (ISPA), 33011 Oviedo, Spain; 32 Paediatric Gastroenterology and Nutrition Unit, Hospital Universitario Central de Asturias, 33011 Oviedo, Spain; 33 Gastroenterology Department, Hospital Clínico de Valencia, 46010 Valencia, Spain; 34 Paediatric Gastroenterology, Hepatology and Nutrition Unit, Hospital Clínico de Valencia, 46010 Valencia, Spain; 35 Inflammatory Bowel Disease Unit, Department of Gastroenterology and Hepatology, Hospital Puerta de Hierro, 28222 Madrid, Spain; 36 Gastroenterology Department, Consorcio Hospital General Universitario de Valencia, 46014 Valencia, Spain; 37 Paediatric Gastroenterology, Hepatology and Nutrition Unit, Consorcio Hospital General Universitario de Valencia, 46014 Valencia, Spain; 38 Gastroenterology, Hepatology and Nutrition Unit, Hospital Universitario Infanta Leonor, 28031 Madrid, Spain; 39 Gastroenterology Department, Hospital Universitario Infanta Leonor, 28031 Madrid, Spain; 40 Gastroenterology Department, Hospital Universitario de Fuenlabrada, 28942 Madrid, Spain; 41 Paediatric Gastroenterology Unit, Hospital Universitario de Fuenlabrada, 28942 Madrid, Spain; 42 Paediatric Gastroenterology, Hepatology and Nutrition Unit, Hospital Clínico Universitario Lozano Blesa, 50009 Zaragoza, Spain; 43 Gastroenterology Department, Hospital Clínico Universitario Lozano Blesa, 50009 Zaragoza, Spain; 44 Gastroenterology Department, Hospital Doctor José Molina Orosa, 35500 Las Palmas, Spain; 45 Gastroenterology Department, Hospital Universitario de Basurto, 48013 Bilbao, Spain; 46 Paediatric Gastroenterology, Hepatology and Nutrition Unit, Hospital de Torrejón, 28850 Madrid, Spain; 47 Gastroenterology Department, Hospital de Torrejón, 28850 Madrid, Spain; 48 Gastroenterology Department, Hospital Universitario Infanta Cristina, 28981 Madrid, Spain; 49 Gastroenterology Department, Centro Consorci Sanitari Terrassa, 08227 Terrassa, Spain; 50 Gastroenterology Department, Hospital de Henares, 28822 Madrid, Spain; 51 Paediatric Gastroenterology, Hepatology and Nutrition Unit, Hospital de Henares, 28822 Madrid, Spain; 52 Gastroenterology Department, Hospital de San Jorge, 22004 Huesca, Spain; 53 Gastroenterology Department, Hospital San Pedro, 28037 La Rioja, Spain; 54 Gastroenterology Department, Hospital Virgen de La Luz, 16002 Cuenca, Spain

**Keywords:** transition, transitional care, inflammatory bowel disease

## Abstract

(1) Background: Transition is a planned movement of paediatric patients to adult healthcare systems, and its implementation is not yet established in all inflammatory bowel disease (IBD) units. The aim of the study was to evaluate the impact of transition on IBD outcomes. (2) Methods: Multicentre, retrospective and observational study of IBD paediatric patients transferred to an adult IBD unit between 2017–2020. Two groups were compared: transition (≥1 joint visit involving the gastroenterologist, the paediatrician, a programme coordinator, the parents and the patient) and no-transition. Outcomes within one year after transfer were analysed. The main variable was poor clinical outcome (IBD flare, hospitalisation, surgery or any change in the treatment because of a flare). Predictive factors of poor clinical outcome were identified with multivariable analysis. (3) Results: A total of 278 patients from 34 Spanish hospitals were included. One hundred eighty-five patients (67%) from twenty-two hospitals (65%) performed a structured transition. Eighty-nine patients had poor clinical outcome at one year after transfer: 27% in the transition and 43% in the no-transition group (*p* = 0.005). One year after transfer, no-transition patients were more likely to have a flare (36% vs. 22%; *p* = 0.018) and reported more hospitalisations (10% vs. 3%; *p* = 0.025). The lack of transition, as well as parameters at transfer, including IBD activity, body mass index < 18.5 and corticosteroid treatment, were associated with poor clinical outcome. One patient in the transition group (0.4%) was lost to follow-up. (4) Conclusion: Transition care programmes improve patients’ outcomes after the transfer from paediatric to adult IBD units. Active IBD at transfer impairs outcomes.

## 1. Introduction

Transition is defined as the intentional and planned movement of adolescents and young adults with chronic medical conditions from children to adult-oriented healthcare systems [1]. In Europe, up to 25% of the patients with inflammatory bowel disease (IBD) are diagnosed at a paediatric age, and a growing incidence of paediatric IBD has been reported [2,3,4].

Transition is a complex, structured and dynamic process in which the patient, the parents, the gastroenterologist and the paediatrician must be involved. The agents involved in this process may have different perspectives regarding the management of the disease, and the transition programme allows identification and solution of these differences [5].

During the transition, it is essential to guarantee the continuity of medical assistance from paediatric to adult IBD units [6]. Recently, some clinical practice guidelines and consensus recommendations on transition in IBD have been published [5,7,8,9,10]. However, the majority of their recommendations are based mainly on scientific evidence deemed as “weak” due to the limited number of patients included in the studies and the difficulty of finding an appropriate definition for a “successful transition” [5,7,11]. Regarding this topic, some possible transition models have been proposed [5] and, although there is no evidence suggesting that one model is superior to the others, there is a consensus on the fact that there should be a transition process in chronic diseases, particularly in IBD. Despite certain evidence of the usefulness of the transition programmes in IBD [12,13], their implementation is not yet established in all the paediatric and adult IBD units.

We therefore designed a multicentre and collaborative study between paediatric gastroenterologists and adult gastroenterologists in order to assess the benefit of transition programmes in a real-world setting. The aim of our study was to evaluate the benefit of a planned and structured transition programme for the transfer of IBD patients from paediatric to adult IBD units. We also aimed to assess the prevalence and the different subtypes of transition programmes in Spain and to identify the possible predictive factors of *poor clinical outcome* and loss to follow-up after the transfer.

## 2. Materials and Methods

### 2.1. Study Design and Selection of Participating Centres

A retrospective, multicentre, noninterventional study was carried out in Spain. IBD units that had identified referral paediatricians and accepted to participate in the project were included. Centres with structured transition and others in which the transition programme was not implemented (and patients were just transferred from the paediatric to the adult IBD unit) were included and served as comparators.

The project was assessed by a scientific committee, involving experienced paediatricians on transition in IBD and leaders of the Spanish Society for Paediatric Gastroenterology, Hepatology and Nutrition (SEGHNP). The Spanish Group on Crohn’s Disease and Ulcerative Colitis (GETECCU) evaluated and accepted the study protocol. The study was conducted according to the ethical principles of the Declaration of Helsinki. The study protocol was approved by the Drug Research Ethics Committee (Comité de Ética de la Investigación con Medicamentos) at the Hospital Universitario de La Princesa in Madrid, Spain.

### 2.2. Patient Population

IBD patients diagnosed according to the Porto criteria of the European Society for Paediatric Gastroenterology Hepatology and Nutrition (ESPGHAN) [14] who were transferred from the paediatric to the adult IBD unit from March 2017 to March 2020 were included. Patients with less than one year of follow-up in paediatric care and IBD patients transferred by a different route than the referral paediatric gastroenterologist of the adult IBD unit were excluded. Patients were analysed in two groups depending on whether or not they had undergone a structured transition.

### 2.3. Study Outcomes

The primary outcome was poor clinical outcome, defined as any (at least one) of the following events: (1) Need for hospitalisation due to an IBD flare; (2) Need for surgery due to IBD activity or complications of the IBD; and (3) IBD recurrence/flare-up that required a change in treatment (either intensification of the treatment or change to a different drug and addition of another therapy). The change in the treatment planned by the IBD team during the joint visits and performed immediately after transfer (in the first visit with the adult gastroenterologist) was not considered as a poor clinical outcome.

### 2.4. Data Collection

All clinical data were extracted from the medical records of each participating hospital. Paediatric gastroenterologists identified the patients who finished the follow-up in paediatric care in their medical records (because they had reached the age to be referred to the adult IBD unit) and verified if the patients met the inclusion criteria. The paediatric gastroenterologist also completed the IBD information for each patient until the date of discharge from paediatric care. The adult gastroenterologist included the information of the variables of interest during the first year after the transfer from the paediatric clinic and detected the patients who were lost to follow-up. Moreover, the gastroenterologist identified in their medical records which patients should have been transferred from the referring paediatric gastroenterologist.

The following clinical variables were included in the study: sex, age at IBD diagnosis, smoking history, family history of IBD, disease extension according to the Paris classification [15], presence of perianal disease, psychiatric comorbidities, extraintestinal manifestations (EIMs) and immune-mediated diseases, medical treatments received (exclusive enteral nutrition (EEN), aminosalicylates, corticosteroids, immunomodulators and biologic drugs) and surgical procedures due to an IBD flare or IBD complication. We compiled information from the radiological examinations (magnetic resonance enterography (MRE) and abdominal ultrasound), endoscopic evaluation and biologic markers (albumin, C-reactive protein or faecal calprotectin) when available. We also collected data about the transition programme: starting date, transition place, number of joint visits, transition duration and the professionals involved in the transition. Patients were followed-up until 12 months after transfer.

Study data were collected and managed using the Research Electronic Data Capture (REDCap) electronic data capture tools hosted by the Spanish Gastroenterology Association (AEG). REDCap is a secure, web-based software platform designed to support data capture for research studies, providing (1) an intuitive interface for validated data capture; (2) audit trails for tracking data manipulation and export procedures; (3) automated export procedures for seamless data downloads to common statistical packages; and (4) procedures for data integration and interoperability with external sources [16,17]. Data were remotely monitored to assess data quality.

### 2.5. Definitions

#### 2.5.1. Transition-Related Definitions

- Transition: Intentional and planned movement of adolescents and young adults suffering from chronic physical and medical conditions from child- to adult-oriented healthcare systems. We defined transition as a structured process involving the gastroenterologist, the paediatrician and a programme coordinator, as well as the parents and the IBD patient. At least one joint visit should have been carried out by those involved in the transition process.
- Transfer: Moment at which the medical attention was carried out by the gastroenterologist. We defined the transfer as the date of discharge from the paediatric clinic.

#### 2.5.2. Clinical Definitions

- Type of hospital: Hospitals were clustered in conglomerates, taking into account different variables such as provision, offer of services, activity, complexity or teaching, which established the following three categories: (1) first-level hospital (less than 150 beds on average, hardly any high-tech resource and low complexity); (2) second-level hospital (of average size around 500 beds, medium complexity and some teaching activity); and (3) third-level hospital (large referral hospitals with great structural weight, full offer of services, great teaching and assistance activity and high complexity).
- Smoking status: Patients were considered “smokers” if they smoked more than seven cigarettes per week for at least six months and had smoked at least one cigarette in the six months prior to transfer. Patients were considered “ex-smokers” if they quit smoking at least six months before transfer. Patients were considered “non-smokers” if they never smoked or did so in very small amounts or occasionally. The smoking status was also categorised 12 months after transfer [18].
- IBD location and phenotype were defined according to the Paris classification [15].
- IBD activity: The Harvey–Bradshaw Index (HBI) [19] for Crohn’s disease (CD) and the partial Mayo score [20] for ulcerative colitis (UC) were used in the gastroenterologist setting. On the other hand, the Paediatric CD Activity Index (PCDAI) or the weighted PCDAI (wPCDAI) for CD [21] and the Paediatric UC Activity Index (PUCAI) [22] for UC were used in the paediatric setting. When endoscopy or radiological examination was available, the severity was graded as quiescent, mild, moderate or severe by local investigators.
- Biologic markers: Levels of C-reactive protein and faecal calprotectin were considered as high when >0.5 mg/dL and >250 µg/g, respectively.
- Patients lost to follow-up were defined as those who did not attend the gastroenterology unit during 12 months after transfer.

### 2.6. Statistical Analysis

Patient’s characteristics were analysed according to the presence or absence of a structured transition programme. For categorical variables, percent values and their 95% confidence intervals (CIs) were calculated. For continuous variables, arithmetic mean and the standard deviation, or the median and the interquartile range (IQR), were calculated depending on whether or not they were normally distributed. Categorical variables were compared using the chi-square test (χ^2^) and quantitative variables using the appropriate test (Student’s t and Wilcoxon) depending on whether their values followed a normal distribution or not.

Poor clinical outcome was established as a dependent variable. Independent variables were those that achieved statistical significance in the univariate study or those that were clinically relevant. Variables associated with poor clinical outcome were analysed by the Kaplan–Meier method, in which patients were censored at the time of poor clinical outcome for any reason. Any differences between survival curves were evaluated using the log-rank test. Stepwise multivariable analysis using the Cox regression model was used to investigate factors potentially associated with poor clinical outcome. Patients lost to follow-up were included in the statistical analysis. A subanalysis was carried out exclusively including patients undergoing transition and who were in remission at the time of transfer to the adult IBD unit. In the log-rank test and in the multivariable analysis, *p* values of <0.05 were considered as statistically significant.

## 3. Results

### 3.1. Patient Population

**Figure 1:**
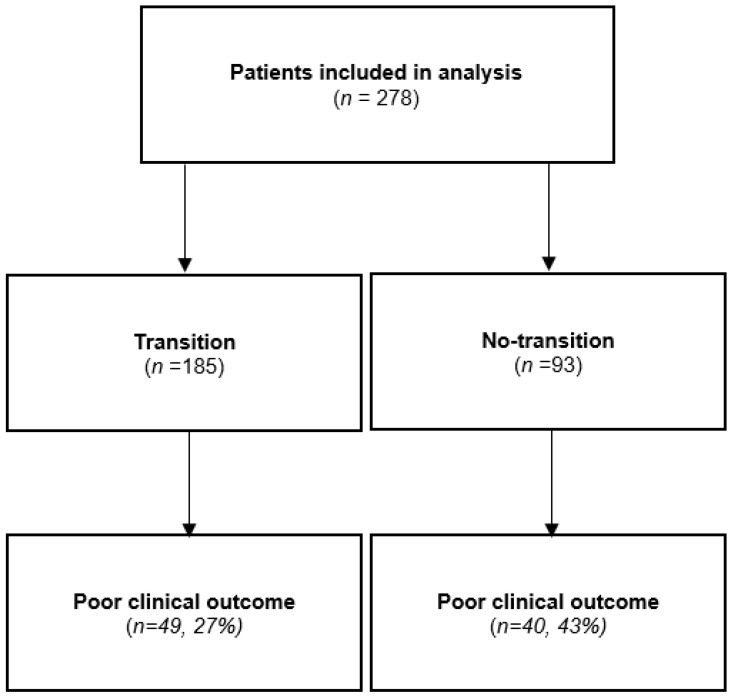
Flow chart of the study population.

A total of 278 patients met the inclusion criteria and were included. Overall, 185 (67%; 95% CI = 61–72) underwent a structured transition and 93 (33%; 95% CI = 28–39) were just transferred from the paediatric to the adult IBD unit (Figure 1).

Patient demographics and baseline characteristics are summarised in Table 1. Family history of IBD and psychiatric comorbidity were more commonly reported in the transition group. Median age at diagnosis was 12 years in both groups, and approximately 60% of the patients were male. Median disease duration at transfer were 54 and 51 months in the transition and in the no-transition group, respectively. In the transition group, 57% of the patients had CD (vs. 56% of the patients in the no-transition group). The majority of CD patients had ileocolonic involvement, and inflammatory behaviour was the most common one (Table 1).

### 3.2. Characteristics of the Participating Hospitals

A total of 34 centres participated in the study. Overall, 22 (65%; 95% CI = 48–78) centres performed a structured transition, and in 12 (35%; 95% CI = 21–52), the transition programme was not implemented. The majority of the centres with an implemented transition programme were large hospitals with medical doctors focused on IBD. Paediatric and gastroenterology IBD units were predominant in the transition group (62% vs. 8%, *p* = 0.001 and 100% vs. 67%, *p* = 0.004, respectively). The mean number of joint visits during transition were 1.5, and the mean duration of the transition was approximately six months. The IBD nurse was involved in the transition programme in 62% of the cases. Characteristics of the participating hospitals are summarised in Table 2.

### 3.3. Clinical Outcomes

Of the 278 patients included, 89 (32%; 95% CI = 27–38) had a *poor clinical outcome* within one year after transfer: 49/185 (27%; 95% CI = 21–33) in the transition group and 40/93 (43%; 95% CI = 33–53) in the no-transition cohort (*p* = 0.005). The median time for a *poor clinical outcome* was 9.3 months (95% CI = 8–10) in the no-transition group and 10.4 months (95%CI 10–11) in the transition cohort (*p* > 0.05).

Regarding outcomes before transfer, prior emergency visits were reported in 47% of the patients in the no-transition group vs. 31% of the patients in the transition cohort (*p* = 0.007). In total, 47% of the patients in the transition group needed hospitalisation vs. 42% of the patients in the no-transition cohort (*p* > 0.05), and corticosteroid treatment was reported in 59% in the no-transition cohort vs. 49% in the transition group (*p* > 0.05). Approximately 80% of patients had received immunosuppressants, and half of the patients had needed biologic drugs (54% in the transition group vs. 41% in the no-transition cohort, *p* = 0.038) (Table 3).

Within one year after transfer, 36% of the patients in the no-transition group had an IBD flare, requiring intensification of the treatment or change to another drug (*p* = 0.018). Emergency visits were reported in 17% of the patients in the no-transition group vs. 10% in the transition cohort (*p* > 0.05). Ten percent of the patients in the no-transition group needed a hospitalisation vs. three percent in the transition cohort (*p* = 0.025). Use of corticosteroids was reported in 15% of the patients in the no-transition group vs. 9% in the transition cohort (*p* = 0.002). The outcomes of interest were not affected by the presence or absence of a paediatric or gastroenterology IBD unit (Table 3).

In eight patients, the paediatric gastroenterologist and the adult gastroenterologist agreed on an elective change in the treatment that was carried out immediately after the transfer: six patients (75%) in the transition group and two patients (25%) in the no-transition cohort. The changes in the treatment for the IBD were that four patients started a new drug, two patients modified the dose of the treatment received, one patient stopped the treatment for IBD and one patient modified and stopped the treatment during the follow-up. All these changes were not considered as *poor clinical outcome* in our study.

Regarding the patients in remission, 150 patients (81%) in the transition group and 75 patients (81%) in the no-transition cohort had quiescent disease at their first referral to the adult IBD unit (*p* > 0.05). Regarding the transitioned patients in remission, 28 patients (19%; 95% CI = 13–26) had a *poor clinical outcome* during the follow-up: 2 patients (1%) required surgery, 2 patients (1%) were hospitalised due to an IBD flare and 26 patients (17%) needed a change in the treatment because of a flare of the disease. On the other hand, 24 patients in the no-transition group in clinical remission at transfer (32%; 95% CI = 23–43) had a *poor clinical outcome* during the follow-up: 6 patients (8%) were hospitalised because of an IBD flare and 18 patients (24%) needed a change in the treatment for the IBD (*p* < 0.001).

### 3.4. Predictive Factors of Poor Clinical Outcome after the Transfer

**Figure 2:**
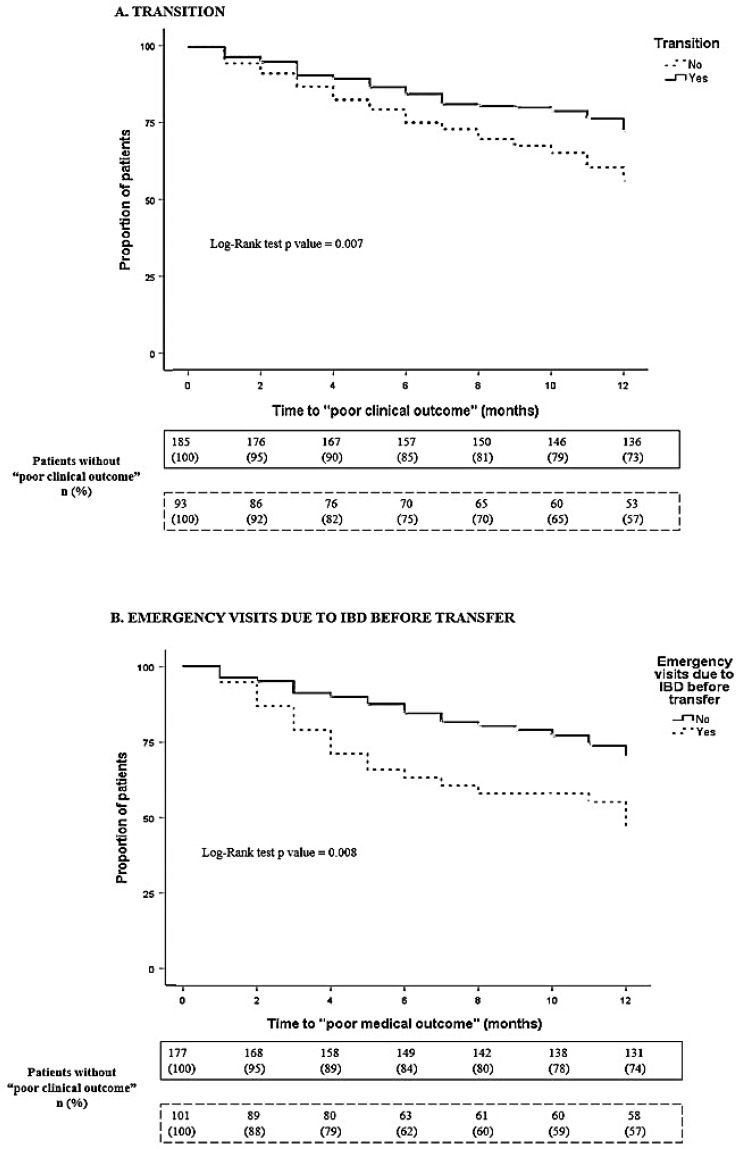

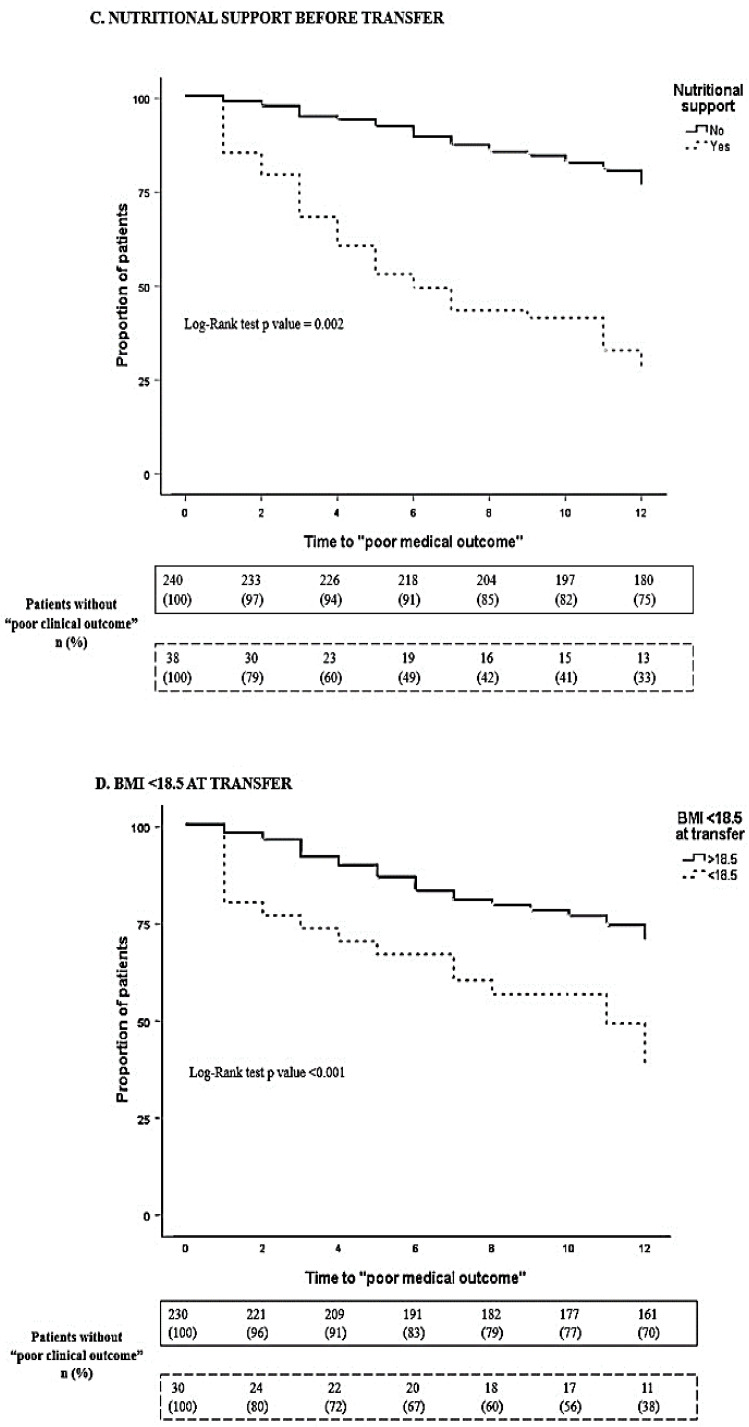

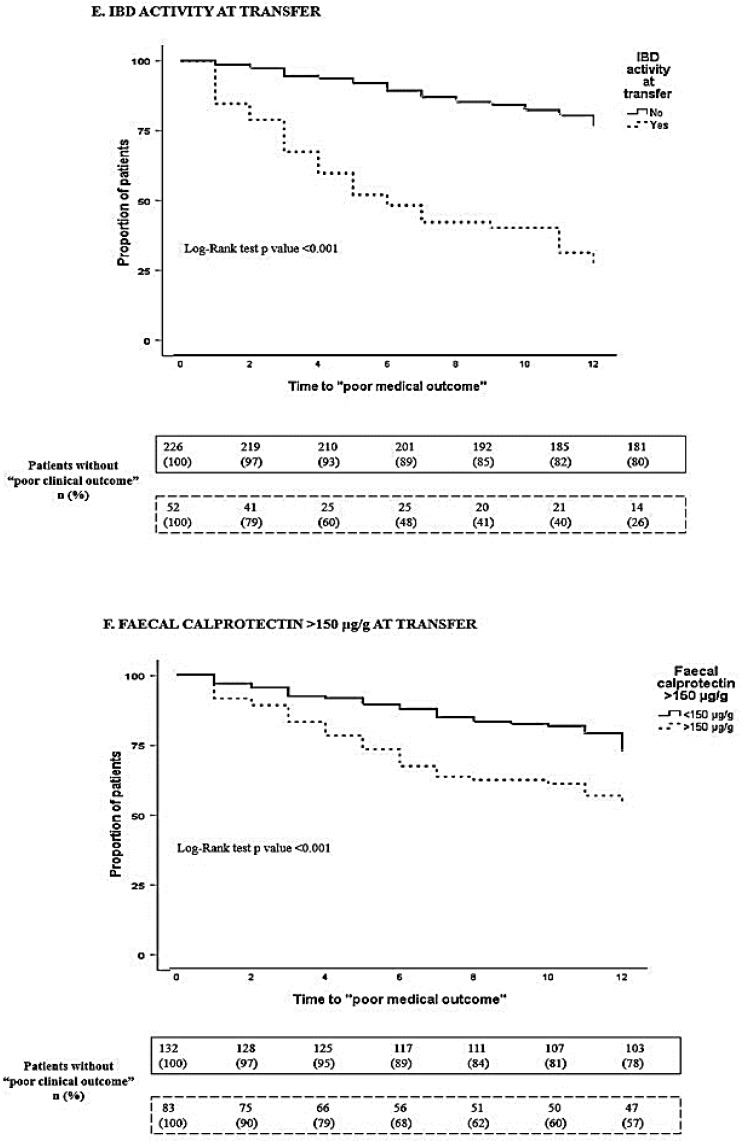

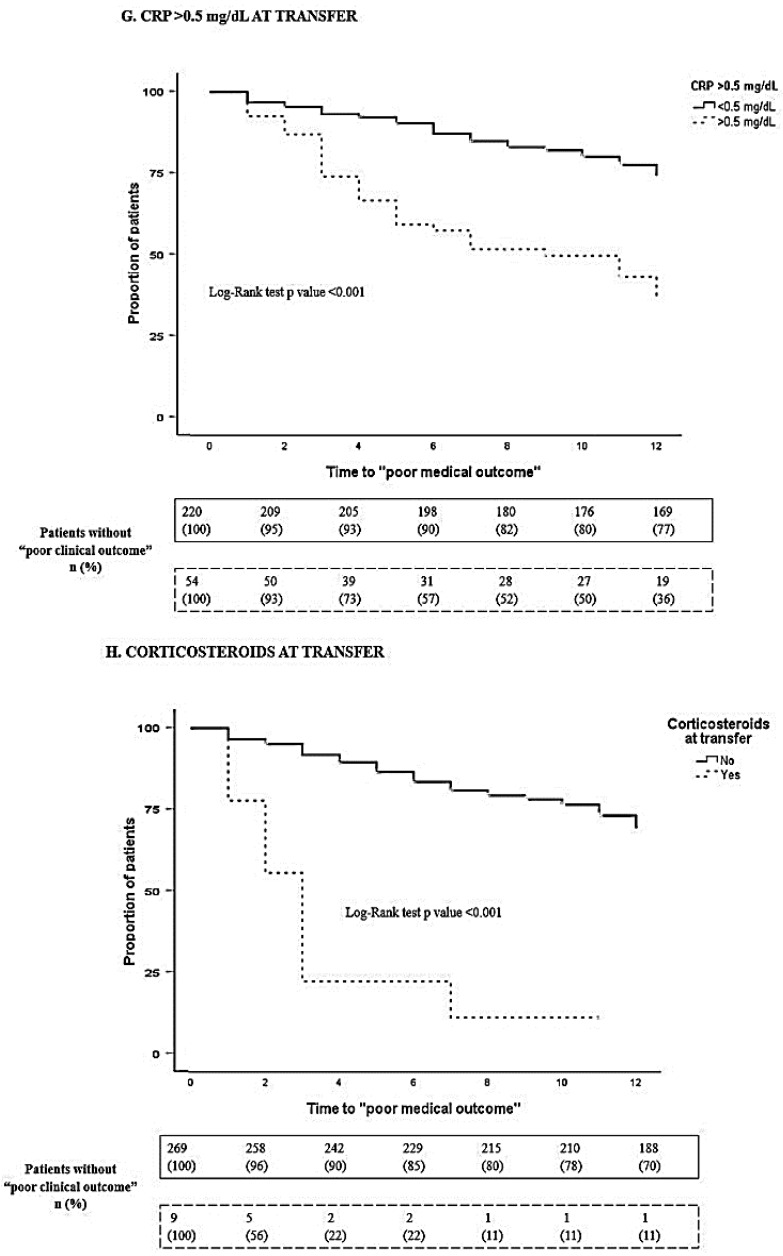
Variables associated with poor clinical outcome. Variables associated with poor clinical outcome were analysed by Kaplan–Meier: (**A**) transition; (**B**) emergency visits due to an IBD flare before transfer; (**C**) nutritional support before transfer; (**D**) BMI < 18.5 at transfer; (**E**) IBD activity at transfer; (**F**) faecal calprotectin > 250 µg/g at transfer; (**G**) C-reactive protein > 0.5 mg/dL at transfer; (**H**) corticosteroids at transfer. Abbreviations: BMI: body mass index; IBD: inflammatory bowel disease.

In the univariate analysis, the variables associated with a *poor clinical outcome* were the absence of structured transition (*p* = 0.007); emergency visits due to an IBD flare before transfer (*p* = 0.008); nutritional support before transfer (*p* = 0.002); body mass index (BMI) < 18.5 at transfer (*p* < 0.001); IBD activity at transfer (*p* < 0.001); faecal calprotectin > 250 µg/g at transfer (*p* < 0.001); C-reactive protein > 0.5 mg/dL at transfer (*p* < 0.001); and treatment with corticosteroids at transfer (*p* < 0.001) (Figure 2). Age at transfer, psychiatric comorbidity and family history of IBD were included in the univariate analysis but did not achieve statistical significance.

In the multivariable analysis, the lack of structured transition, IBD activity at transfer, BMI < 18.5 and corticosteroid treatment at transfer were predictive factors associated with a *poor clinical outcome* (Table 4).

### 3.5. Patients Lost to Follow-Up

In total, 12 patients (4%; 95% CI = 2–7) did not complete the 12 months of follow-up: 11/185 (0.06%; 95% CI = 0.03–0.11) in the transition group and 1/93 (0.01; 95% CI = 0.002–0.06) in the no-transition cohort (*p* > 0.05).

Regarding the patients in the transition group (11/185), one patient (1/185; 0.5%) refused to attend the IBD unit after transfer. He was an 18-year-old male patient with previous diagnosis of ileocolonic CD treated with azathioprine from the date of diagnosis. After transfer, he also decided to stop azathioprine, remaining without any IBD flare during the 12 months after transfer. The remaining 10 patients in the transition group attended the first visit in the IBD unit, but they missed the following visits according to the clinical practice of their centre. Among them, two patients were hospitalised at one year after transfer.

Finally, in the no-transition group, one patient was lost to follow-up and was hospitalised because of an IBD flare within 12 months after transfer.

## 4. Discussion

In the present multicentre study, the benefit of a structured transition programme from paediatric to adult IBD units has been evaluated. To our knowledge, this is the largest cohort of patients in which this outcome has been assessed. Fewer IBD flares, corticosteroid treatments, emergency visits and hospitalisations due to an IBD complication were observed among paediatric patients undergoing structured transition compared with those without transition.

Our results are consistent with other observational studies conducted in patients transferred to the adult gastroenterologist. Firstly, a British cohort reported that structured transition was associated with positive outcomes as the transition group was more likely to be corticosteroid-free one year after transfer and had less emergency visits [23]. Testa et al. also concluded that there was a significant reduction in the number of flares and hospitalisations within the 12 months after a structured transition [24]. Also, the proportion of patients in clinical remission after transfer was higher in the transition vs. in the no-transition group in an Hungarian cohort (95% vs. 65%; *p* = 0.037) [25], and similar data were reported in a Dutch study with a two-year follow-up after the transfer [26].

In our study, achieving remission at transfer was key for the outcomes of IBD patients, as other studies have previously reported [7,27,28]. Schutz et al. described that the proportion of patients in remission at transfer was lower in the no-transition group (63% vs. 88%); in this subgroup, IBD complications were also significantly higher at one year after transfer (64% vs. 21%) [29]. Although the proportion of patients in remission at transfer in our population was balanced between both groups (transition and no-transition), the proportion of IBD patients in remission at transfer with poor clinical outcome during the follow-up was higher in the no-transition group. This finding emphasises that a structured transition is essential in all IBD patients in our daily practice.

One of the issues regarding the benefit of transition is the definition of “successful transition”, which is still not clear because of the lack of comparative studies. Accordingly, nowadays, no specific model can be recommended yet [7,8,9]. Similarly, the number of necessary joint visits between the paediatric and the adult gastroenterologists remains unknown. Erős et al. recently published the protocol of the first randomised controlled trial aiming to provide evidence on the superiority of joint visits compared with standard transitional care in IBD [30]. In our country, the implemented model for transition between paediatric and adult gastroenterologists consists in joint visits. This model has been reported in other studies focused on transitional care [25,26,29] and follows the recommendations of the European Crohn’s and Colitis Organisation (ECCO) [7]. Based on our results, at least one joint visit could be appropriate to improve the patients’ outcomes. Moreover, the European consensus highlights that patients’ self-reported health efficacy appears to improve with age. Accordingly, in our cohort, patients in the transition group were transferred at an older age than those in the no-transition group.

Furthermore, the ECCO consensus suggests that the most commonly recommended transition models in IBD are based on a collaborative approach between paediatric and adult IBD teams, as well as a programme coordinator (in most of the cases an IBD nurse) [7]. IBD nurses are frequently the reference for patients and caregivers, and they also provide an invaluable resource for patients [6,31]. Having a dedicated outpatient clinic with nursing care is one of the quality indicators of GETECCU [32] and, in our study, of the 22 hospitals with transition, 20 (91%) had a nurse in the IBD team. On the other hand, the proportion of nutritionists involved in the transition programme in our country was low (5%). In our study, a low BMI at transfer was associated with poor clinical outcomes after transfer. Therefore, based on our results, it could be of great interest to have a multidisciplinary team, including a nutritionist, during the transition programme.

Due to the rising incidence of paediatric IBD, the number of IBD patients transferred to adult units will increase over the coming years. Our study has demonstrated that a structured transition programme improves the outcomes of IBD patients. However, resources and training for transitional care in our daily practice are scarce and, therefore, a relevant proportion of institutions do not have an established transition programme (35% of the hospitals in our study). These barriers have recently been acknowledged in a structured survey in Spain [33]. This survey was distributed to paediatric and adult gastroenterologists, and the response rate was only 12%, and it was significantly higher among paediatric vs. adult gastroenterologists. To date, there are no specific clinical guidelines for transition in IBD in Spain, unlike in other countries [5,9,10]. Therefore, based on our results, developing a clinical practice guideline about transitional care in our country could improve the management of IBD patients in this critical phase. Moreover, an appropriate transitional care should be considered as a healthcare objective that requires the support of the health system at all times. Due to the hindrances that can occur during the implementation of a transition programme, medical education about transition is key, and experienced IBD-teams should train those who have not implemented a transition programme.

The limitations of the present study are mainly those associated with its retrospective design, including the potential for missing data in clinical records. This may be a possible explanation for the low proportion of patients lost to follow-up. However, we made an effort to investigate the evolution of patients that did not attend the adult IBD unit. Also, it is worth highlighting that patients undergoing structured transition in our cohort were more likely to be clustered in larger centres with established paediatric and adult IBD units compared with the no-transition patients. Even though we also included small centres and hospitals in which the transition programme was not implemented, most of the paediatric and adult gastroenterologists involved were focused on IBD. In addition, IBD units of the no-transition groups had a referral paediatrician and, therefore, our results may not be fully extrapolated to all centres without transition.

Our collaborative study has several strengths. First, this is the largest real-world study evaluating the benefit of a transition programme in IBD patients. The retrospective design of the study allowed us to evaluate the management of transferred patients from the paediatric to the adult IBD units in a real-world setting. Moreover, flares were detected not only by hospitalisations or therapy escalation but also by objective activity index, evaluated by the Mayo score or the HBI. Finally, our study investigated the predictors of poor clinical outcome, which are key factors in our daily clinical practice.

In conclusion, our study demonstrates the benefit of a transition programme in IBD. Transition care programmes improve patients’ outcomes after the transfer from paediatric to adult IBD units. Active IBD at transfer impairs patient’s outcomes.

## Figures and Tables

**Table 1:** Patient baseline demographics and disease characteristics.

		Transition Group (*n* = 185)	No-Transition Group (*n* = 93)	*p* Value
Gender male, *n* (%)		115 (62)	54 (58)	N.S.
Age at diagnosis, years (median, IQR)		12 (1–17)	12 (3–17)	N.S.
Age at transfer, years (median, IQR)		17 (14–20)	16 (14–20)	<0.01
Disease duration at transfer, months (median, IQR)		54 (12–211)	51 (12–171)	N.S.
Family history of IBD, *n* (%)		26 (14)	24 (26)	<0.05
Smokers, *n* (%)		11 (6)	3 (3)	N.S.
EIM, *n* (%)		30 (16)	16 (17)	N.S.
Psychiatric comorbidity, *n* (%)		37 (20.1)	8 (8.7)	<0.05
IBD type, *n* (%)		CD: 106 (57) UC: 73 (40) IBD unclassified: 6 (3)	CD: 52 (56) UC: 38 (41) IBD unclassified: 3 (3)	N.S.
UC † (*n* = 120)	UC extent, *n* (%)	Ulcerative proctitis: 13 (17) Left-sided UC: 13 (17) Extensive UC: 11 (14) Pancolitis: 42 (52)	Ulcerative proctitis: 3 (7) Left-sided UC: 8 (19) Extensive UC: 8 (19) Pancolitis: 22 (55)	N.S.
	UC severity, *n* (%)	Never severe: 51 (65) Ever severe: 28 (35)	Never severe: 30 (73) Ever severe: 11 (27)	N.S.
CD (*n* = 158)	CD location, *n* (%) ‡	Distal 1/3 ileal ± limited cecal disease: 25 (24) Colonic: 8 (8) Ileocolonic: 66 (62) Upper disease proximal to Treitz: 26 (25) Upper disease distal to Treitz: 11 (10)	Distal 1/3 ileal ± limited cecal disease: 7 (14) Colonic: 9 (17) Ileocolonic: 36 (69) Upper disease proximal to Treitz: 11 (21) Upper disease distal to Treitz: 1 (2)	N.S.
	CD behaviour, *n* (%)	Inflammatory: 86 (81) Stricturing: 16 (15) Penetrating: 3 (3) Penetrating and stricturing: 1 (1)	Inflammatory: 47 (90) Stricturing: 2 (4) Penetrating: 3 (6) Penetrating and stricturing: 0 (0)	N.S.
	Growth delay, *n* (%)	20 (19)	6 (12)	N.S.
	Perianal disease, *n* (%)	32 (30)	10 (19)	N.S.

IBD: inflammatory bowel disease; CD: Crohn’s disease; UC: ulcerative colitis; EIM: extraintestinal manifestations; IQR: interquartile range; N.S.: nonstatistically significant. † We included both UC and IBD unclassified. ‡ Patients may belong to more than one group.

**Table 2:** Characteristics of the participating hospitals.

	Transition Group (*n* = 22)	No-Transition Group (*n* = 12)	*p* Value
Third-level hospital (paediatric unit), *n* (%)	17 (77)	6 (50)	N.S.
Third-level hospital (gastroenterology unit), *n* (%)	16 (73)	6 (50)	N.S.
Paediatric IBD unit (yes), *n* (%)	15 (68)	1 (8)	<0.01
Gastroenterology IBD unit (yes), *n* (%)	22 (100)	8 (67)	<0.01
IBD patients followed-up in paediatric units (median, IQR)	28 (43)	15 (70)	N.S.
IBD patients followed-up in gastroenterology units (median, IQR)	1700 (1126)	1075 (1250)	N.S.
Transition programme starting date, *n* (%)	<2010: 1 (4) 2010–2015: 8 (37) 2016–2020: 12 (55) >2020: 1 (4)	NA	NA
Transfer type (no-transition), *n* (%)	NA	IBD specialist: 11 (92) General gastroenterologist: 1 (8)	NA
Number of patients transferred per year (median, IQR)	3 (3)	NA	
Transition place, *n* (%)	Paediatric clinic: 3 (14) Gastroenterology clinic: 10 (45) Both clinic: 9 (41)	NA	NA
Number of joint visits (mean ± SD)	1.5 ± 0.8	NA	NA
Transition duration (median, IQR), months	6 (6)	NA	NA
Professionals involved in transition, *n* (%)	Paediatrician and gastroenterologist: 21 (100) Paediatric nurse: 7 (33) Gastroenterology nurse 13 (62) Nutritionist: 1 (5) Social worker: 0 (0)	NA	NA
Paediatrician focused on IBD, *n* (%)	19 (91)	NA	NA
Gastroenterologist focused on IBD, *n* (%)	21 (100)	NA	NA

IBD: inflammatory bowel disease; SD: standard deviation; IQR: interquartile range; NA: not applicable; N.S.: nonstatistically significant.

**Table 3:** Clinical outcomes.

OUTCOMES BEFORE TRANSFER			
	Transition Group (*n* = 185)	No-Transition Group (*n* = 93)	*p* Value
Emergency visits, *n* (%)	57 (31)	44 (47)	<0.01
Hospitalisations, *n* (%)	77 (42)	44 (47)	N.S.
Surgeries, *n* (%)	23 (12)	7 (8)	N.S.
IBD-related treatments, *n* (%)	Aminosalicylates: 94 (51) Topic treatment: 50 (27) Corticosteroids: 91 (49) IMM: 145 (78) Biologic drugs: 100 (54) EEN: 58 (31) Apheresis: 5 (3) Nutritional support: 21 (11)	Aminosalicylates: 59 (63) Topic treatment: 28 (30) Corticosteroids: 55 (59) IMM: 76 (82) Biologic drugs: 38 (41) EEN:31 (33) Apheresis: 2 (2) Nutritional support: 17 (18)	<0.05 N.S. N.S. N.S. <0.05 N.S. N.S. N.S.
**OUTCOMES ONE YEAR AFTER TRANSFER**			
IBD flare, *n* (%)	41 (22)	33 (36)	<0.05
Emergency visits, *n* (%)	18 (10)	16 (17)	N.S.
Hospitalisations, *n* (%)	6 (3)	9 (10)	<0.05
Surgeries, *n* (%)	2 (1)	3 (3)	N.S.
IBD-related treatments, *n* (%)	Aminosalicylates: 74 (40) Topic treatment: 20 (11) Corticosteroids: 9 (5) IMM: 95 (51) Biologic drugs: 98 (53) Nutritional support: 2 (1)	Aminosalicylates: 44 (47) Topic treatment: 7 (8) Corticosteroids: 15 (16) IMM: 58 (62) Biologic drugs: 44 (47) Nutritional support: 1 (1)	N.S. N.S. <0.01 N.S. N.S. N.S.

IBD: inflammatory bowel disease; IMM: immunomodulators; EEN: exclusive enteral nutrition; N.S.: nonstatistically significant.

**Table 4:** Multivariable analysis of factors associated with poor clinical outcome.

Factor	HR	95% CI
No transition	2.1	1.3–3.3
IBD activity at transfer	4.9	3.1–7.9
BMI < 18.5 at transfer	1.9	1.1–3.3
Corticosteroids at transfer	4.9	2.2–10.9

IBD: inflammatory bowel disease; BMI: body mass index; HR: hazard ratio; CI: confidence interval.

## Data Availability

The data underlying this article will be shared on reasonable request to the corresponding author.

## Abbreviations

Inflammatory bowel disease (IBD); Crohn’s disease (CD); ulcerative colitis (UC); immunomodulator (IMM); body mass index (BMI); Hazard Ratio (HR); confidence interval (CI).
